# Presence of Psychotic Spectrum Symptoms Before Age 12 in Schizophrenia Patients: A Retrospective Study on Clinical Implications for Early Detection and Intervention

**DOI:** 10.3390/brainsci15030311

**Published:** 2025-03-15

**Authors:** Pietro Carmellini, Alessandro Cuomo, Annarita Vignapiano, Francesco Monaco, Simone Pardossi, Bernardo Firenzuoli, Andrea Fagiolini

**Affiliations:** 1 Department of Molecular and Developmental Medicine, Division of Psychiatry, University of Siena School of Medicine, 53100 Siena, Italy; alessandrocuomo86@gmail.com (A.C.); pardossisimone@gmail.com (S.P.); bernardo.firenzuoli@gmail.com (B.F.); andreafagiolini@gmail.com (A.F.); 2 Department of Mental Health, Azienda Sanitaria Locale Salerno, 84125 Salerno, Italy; annarita.vignapiano@gmail.com (A.V.); fmonaco1980@gmail.com (F.M.); 3 European Biomedical Research Institute of Salerno (EBRIS), 84125 Salerno, Italy

**Keywords:** prodromal symptoms, schizophrenia, early detection, psychotic spectrum

## Abstract

**Background/Objectives**: Schizophrenia is a severe psychiatric disorder, with onset typically occurring in late adolescence or early adulthood. Early identification of psychotic symptoms, especially those occurring before age 12, has been linked to better long-term outcomes. This study aims to assess the presence of psychotic spectrum symptoms before the age of 12 in adult schizophrenia patients and explore their clinical implications for early detection and intervention. **Methods**: This retrospective, observational study included 170 adult patients diagnosed with schizophrenia, confirmed by the SCID-5. Patients were recruited from the University of Siena Medical Center and completed the modified lifetime version of the Psychotic Spectrum Self-Report (PSY-SR) questionnaire, which assessed the onset of specific psychotic symptoms before and after age 12. Symptom severity was evaluated using the Brief Psychiatric Rating Scale (BPRS) and the Clinical Global Impression Scale (CGI). This study also examined the impact of the duration of untreated psychosis (DUP) on symptom severity. **Results**: In our cohort, 21% of patients exhibited prodromal symptoms before age 12 (95% CI: 15–27%). Prodromal symptoms were linked to a 9.53-point increase in the BPRS scores (*p* = 0.0478) and a 0.50-point increase in the CGI scores (*p* = 0.0347). The age of symptom onset negatively correlated with the BPRS scores (*p* < 0.0001), with each year of delay resulting in a 1.33-point decrease. The DUP correlated significantly with both the BPRS (ρ = 0.97) and CGI scores (ρ = 0.94). The multivariate analysis revealed that a longer DUP was associated with significant increases in both scores: a 27.16-point increase in the BPRS (*p* < 0.0001) for a moderate DUP and a 67.51-point increase (*p* < 0.0001) for a severe DUP. The CGI scores increased by 1.11 points with a moderate DUP and 3.17 points with a severe DUP (*p* < 0.0001). However, the interaction between the DUP and prodromal symptoms at age 12 was not significant, indicating similar impacts of the DUP regardless of early symptom onset. **Conclusions**: The results support the critical importance of early detection and intervention in schizophrenia. Early psychotic spectrum symptoms, particularly those occurring before age 12, are significant predictors of later severity and functional impairment. This study underscores the value of screening tools like the PSY-SR for identifying prodromal symptoms and facilitating timely intervention. Our findings highlight the need for the early identification of psychotic symptoms, particularly in at-risk populations, to improve long-term outcomes. Intervening before the onset of full-blown psychosis may reduce the severity of schizophrenia and promote better clinical outcomes.

## 1. Introduction

Schizophrenia, affecting an estimated 1% of individuals globally, is a debilitating psychiatric disorder that ranks among the leading causes of disability worldwide [1]. The incidence of schizophrenia rises sharply, peaking between ages 15 and 25 in males. In females, there is an initial peak between ages 15 and 30, followed by a second, less pronounced peak around menopausal age (44–49 years) [2]. Emerging evidence highlights the importance of early identification of psychotic symptoms, particularly those appearing before the age of 12, as they are associated with more severe psychopathology and worse long-term outcomes in schizophrenia [3]. Children exhibiting early-onset symptoms, such as negative symptoms and cognitive impairments, often show greater functional impairment and a heightened severity of illness at the time of diagnosis [4]. The prodromal phase is marked by a constellation of nonspecific symptoms, such as diminished stress tolerance, emotional expressivity, and increased social withdrawal, which are often precursors to more severe psychopathology [5]. These early symptoms, though indicative of a heightened risk for psychosis, are frequently misinterpreted as normative adolescent behaviors, thereby complicating the early identification process [6]. Research has established that the latency between prodromal symptom onset and treatment initiation is inversely related to the severity of schizophrenia at diagnosis [7], and the broad spectrum of schizophrenia symptoms, which can range from hallucinations to cognitive disturbances, has been shown to be more severe with the early onset of the disorder [8] underscoring the importance of early detection and treatment of prodromal symptoms. Clinical staging, which categorizes symptom clusters and remission levels, enhances early intervention by emphasizing that the duration of untreated psychosis (DUP) determines symptom severity at the first episode [9,10]. The DUP, defined as the time between the onset of psychotic symptoms and the initiation of appropriate treatment, is a reliable predictor of both clinical and functional outcomes [11]. The suboptimal outcomes observed in many patients with a prolonged DUP underscore the critical importance of early detection and intervention at the first episode of psychosis. In this context, the spectrum model of psychotic symptoms, which challenges traditional categorical diagnoses, proposes a continuum that spans various psychiatric disorders, including delusions, hallucinations, traits of hypertrophic self-esteem, interpersonal sensitivity, anger-overreactivity, and misperceptions [12,13]. The Structured Clinical Interview for the Psychotic Spectrum (SCI-PSY) [14] aligns with this approach, offering a comprehensive framework for identifying prodromal symptoms within the broader psychotic spectrum [15,16,17,18]. While categorical models focus on fixed diagnostic thresholds, dimensional models allow for a broader understanding of symptom severity and progression, making them particularly useful for early detection and intervention [19]. These findings underscore the need for timely intervention and closer monitoring of prodromal symptoms in children, as early detection can significantly influence the course of the disorder and improve prognosis [20]. Furthermore, family dynamics and parental mental health have been shown to influence the manifestation and progression of psychotic symptoms in early-onset schizophrenia. Research suggests that a family history of mental illness and parental psychosocial stress may exacerbate symptoms, increasing the severity and earlier onset of psychosis. These family-related factors, when coupled with genetic predisposition, underscore the importance of addressing both the individual and familial context in early interventions [21,22]. The primary objective of this study is to assess the presence of prodromal psychotic spectrum symptoms before the age of 12 in a sample of adult patients with schizophrenia using the self-report lifetime version of the SCI-PSY (PSY-SR). The PSY-SR operationalizes prodromal symptoms into specific domains, each reflecting distinct risk factors that enable the identification of early psychotic signs and provide valuable insights into the progression of the disorder before the onset of full psychosis. Additionally, this study aims to evaluate the impact of these early symptoms on the severity of schizophrenia in adulthood. The secondary objective is to assess the influence of the DUP on the severity of schizophrenia in adult patients. This investigation seeks to enhance our understanding of the predictive value of these early symptoms in the context of schizophrenia’s development, potentially informing more effective early intervention strategies.

## 2. Materials and Methods

### 2.1. Study Design and Participants

This was a retrospective, observational study approved by the Ethics Committee of the University of Siena Medical Center (protocol number 26064). A total of 170 patients diagnosed with schizophrenia, confirmed by the SCID-5 clinical version, were consecutively recruited within the Psychiatry Unit of Siena University Hospital and invited to participate. These patients were selected from those who were actively followed at our center and met the inclusion criteria. During their assessment visits, patients provided informed consent and completed the PSY-SR questionnaire. At the same visit, trained psychiatrists assessed the severity of schizophrenia using the Brief Psychiatric Rating Scale (BPRS).

For each specific psychotic spectrum symptom listed in the PSY-SR, the patients were asked to indicate the age of onset. Responses indicating symptom onset before the age of 12 for at least 30 items were considered positive for prodromal symptoms. This cutoff was chosen based on clinical experience and judgment, as there is no universally established threshold for identifying prodromal symptoms. The selection of 30 items was intended to reflect a significant presence of symptoms across multiple domains of the psychotic spectrum; it was considered a clinically relevant and meaningful marker of prodromal symptoms, enabling us to identify individuals at higher risk for developing psychosis.

The inclusion criteria for this study were a diagnosis of schizophrenia, age between 18 and 60 years, and the ability to provide informed consent. The exclusion criteria included an age over 60 years and the presence of a major neurocognitive disorder. To ensure that the participants possessed the necessary cognitive and intellectual capacity to complete the PSY-SR, we excluded individuals with an IQ below 80, as well as those with a history of significant neurodevelopmental disorders, which could have impacted their ability to accurately respond to the questionnaire. Socio-demographic data, clinical characteristics, treatment details, and outcome measures were extracted from the patients’ medical records. The evaluations and completion of the PSY-SR were conducted once during the patients’ visits, with the clinicians guiding the process as needed, ensuring accurate and consistent responses.

### 2.2. Outcome Measures

In this study, the PSY-SR lifetime questionnaire, derived from the SCI-PSY with no significant differences in terms of the content and the structure of the items, was used to assess the prodromal symptoms of psychosis. The PSY-SR is a 164-item questionnaire designed to assess a wide range of psychopathological manifestations associated with psychotic syndromes, schizoid and schizotypal personality disorders, as well as atypical and subthreshold psychotic symptoms experienced throughout an individual’s lifetime. This self-report tool categorizes psychotic symptoms into five domains, including interpersonal sensitivity, misperceptions, paranoid traits, schizoid traits, and typical psychotic symptoms such as hallucinations and delusions. Each item is coded as “yes” or “no,” indicating the presence or absence of a symptom. The total score, with a possible range from 0 to 164, is the sum of the scores across all domains and reflects the severity and breadth of the psychotic spectrum symptoms, with higher scores suggesting a greater presence of these symptoms. Currently, no specific threshold has been established for the total score for any of the instruments. The SCI-PSY’s strong psychodiagnostic properties make it an effective instrument for operationalizing prodromal symptoms, identifying specific risk factors, and enhancing our understanding of the psychotic spectrum and has demonstrated robust psychometric properties, with Kuder–Richardson coefficients greater than 0.50 for all domains, and exceeding 0.70 for 12 out of the 16 domains, reflecting its internal consistency and reliability in assessing early psychotic symptoms [14]. The psychometric properties of the PSY-SR are expected to be comparable to those of the SCI-PSY in consideration of the same structure and content of the SCI-PSY. Moreover, to further ensure the accuracy and validity of the PSY-SR, a trained clinician was present during the completion of the questionnaire to guide the patient and clarify any doubts. This approach helped mitigate potential issues related to the self-report nature of the instrument, ensuring that its psychometric properties and validity were maintained throughout the assessment process.

The severity of schizophrenia in adult patients was assessed using the Brief Psychiatric Rating Scale (BPRS) [23] and the Clinical Global Impression Severity Scale (CGI-S) [24]. The BPRS, a clinician-administered scale, measures the severity of psychiatric symptoms across multiple domains, including positive, negative, and affective symptoms, and is widely used to evaluate treatment outcomes and symptom severity. Cronbach’s alpha for the BPRS has been reported to range from 0.80 to 0.94, demonstrating its strong internal consistency and reliability [25]. The CGI-S, a clinician-rated scale, provides a global measure of the overall severity of the patient’s illness, reflecting their clinical functioning. These scales were employed to determine the impact of prodromal symptoms before age 12 and the duration of untreated psychosis (DUP) on the severity of schizophrenia.

### 2.3. Statistical Analysis

The prevalence of patients with prodromal symptoms, along with 95% confidence intervals (95% CIs), was calculated using the Clopper–Pearson method. The sample size of 170 patients was selected based on statistical power calculations, which ensured that this study had sufficient power to detect meaningful associations between the presence of prodromal symptoms before age 12 and schizophrenia severity. This sample size is typical for studies of similar clinical designs and allows for reliable estimates and subgroup analyses, considering the expected effect size and the desired level of precision. To investigate whether the presence of prodromal symptoms before the age of 12 or the DUP was associated with the BPRS and CGI scores, multivariate linear regression models were implemented. These models adjusted for socio-demographic variables (gender, age, age of symptom onset, marital status, educational level, and occupational status), clinical history (stressful life events and familial history of psychiatric disorders), and medication use (antidepressants, mood stabilizers, and benzodiazepines). Additionally, two linear regression models were applied to evaluate the association between the DUP (categorized as “0 years”, “1–5 years”, “>5 years”) and BPRS and CGI scores. Adjusted mean scores for each DUP category were calculated, and pairwise comparisons between categories were performed using Tukey’s method to control for multiple testing. All tests were two-sided and a *p*-value less than 0.05 was considered as the significance level. All analyses were performed using SAS 9.4 (SAS Institute Inc., Cary, NC, USA).

## 3. Results

### 3.1. Overall Cohort

A total of 170 patients diagnosed with schizophrenia, aged between 18 and 90 years, were included in this study. The prevalence of patients exhibiting prodromal symptoms before age 12 was 21% (95% CI: 15–27%). The distribution of these symptoms was as follows: 21% of patients (35 patients) showed at least 30 items before age 12, 28% (48 patients) exhibited at least 29 items, and 35% (60 patients) had at least 28 items. Furthermore, 42% of patients (71 patients) met the threshold of at least 27 items, while 48% (82 patients) exhibited at least 26 items before the age of 12.

Table 1 and Table 2 provide an overview of the socio-demographic and clinical characteristics of the patients in the cohort, with comparisons between those who experienced prodromal symptoms before age 12 and those who did not. The cohort was mostly composed of males (62%) with a mean age of 51 (SD: 13.49) years.

The median age at symptom onset was 20 (IQR: 17–26), while the median age at the first psychiatric evaluation was 25 (20–31). The time from symptom onset to initial psychiatric assessment had a median of 3 (1–6) years, with 81% experiencing a delay (≥1 year) in diagnosis.

Severity assessments, based on the CGI scale, showed that 58% of patients were rated as moderately ill (CGI < 5), while 42% were classified as severely ill CGI ≥ 5). The median BPRS score was 113.5 (87–148), while the median retrospective self-reported PSY-SR scores at 12 years old were 33 (13–40). A high proportion (71%) reported experiencing a stressful event in their history, and 86 patients (51%) had a family history of mental illness.

### 3.2. Group Differences

In our cohort, 35 patients (21%) exhibited prodromal symptoms before the age of 12, while the remaining 135 (79%) did not report such early symptoms.

Gender distribution was similar across groups, with females representing 40% of patients with prodromal symptoms and 38% of those without (*p* = 0.8095). The mean age was also comparable between groups (49.49 vs. 51.32 years, *p* = 0.4753). In terms of medication use, a significantly lower proportion of patients with prodromal symptoms used antidepressants compared to those without (3% vs. 16%, *p* = 0.0494). Utilization of antipsychotics was universal in both groups (100%), and other medication utilization, including mood stabilizers (MS) and benzodiazepines (BDZ), did not differ significantly.

Table 2 reports the clinical information about the onset and severity of schizophrenia. Patients with prodromal symptoms had a slightly earlier age of onset for schizophrenia symptoms compared to those without (18 [16–27] vs. 20 [17–26] years old), though this was not statistically significant (*p* = 0.2638). The median age at the baseline schizophrenia evaluation was similar across groups (27 [20–31] vs. 25 [20–32] years old, *p* = 0.7765).

Both groups had comparable rates of delayed diagnosis, with over 80% experiencing over 1 year of diagnosis delay (*p* = 0.7753). The median time from symptom onset to the first psychiatric evaluation tended to be longer in the prodromal group versus the non-prodromal group (4 [1–10] vs. 2 [1–6] years), though it was not statistically significant (*p* = 0.0726).

In terms of severity, patients with prodromal symptoms had a slightly higher median CGI score compared to those without (5 [3–6] vs. 4 [3–6]), though this was not statistically significant (*p* = 0.0783). Similarly, in comparing the BPRS scores between the two groups, patients with prodromal symptoms showed a trend toward higher median scores compared to those without prodromal symptoms (131 [89–155] vs. 111 [87–145]), although this difference did not reach statistical significance (*p* = 0.1236).

The retrospective self-reported psychopathology scores indicated a greater symptom burden during childhood in patients with prodromal symptoms, with significantly higher median PSY-SR scores at 12 years old (37 [30–44] vs. 32 [9–38], *p* = 0.0002).

Among the subgroup of patients who experienced prodromal symptoms, hallucinations were reported by 15 patients (42%), while 16 patients experienced delusions (46%), and 4 patients experienced both hallucinations and delusions (12%).

### 3.3. Impact of Prodromal Symptoms at Age 12 on the BPRS and CGI

**Figure 1:**
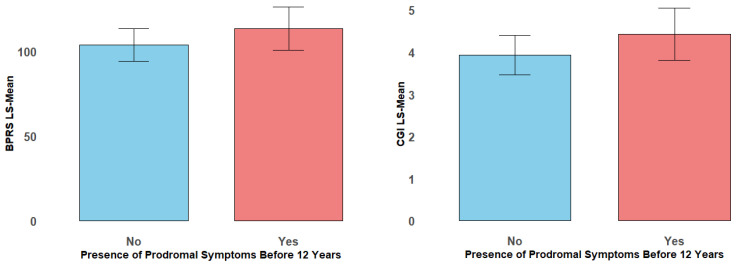
Boxplots illustrating the distribution of BPRS and CGI scores stratified by the presence of prodromal symptoms before 12 years old. The boxes represent the interquartile ranges, with the median indicated by the line within each box.

For the BPRS, the presence of prodromal symptoms before age 12 was linked to a significant increase of 9.53 points (95% CI: 0.09 to 18.96, *p* = 0.0478). Additionally, the age of symptom onset showed a strong negative association, with the BPRS scores decreasing by 1.33 points for each additional year of delay in symptom onset (*p* < 0.0001). For the CGI, the presence of prodromal symptoms before age 12 was significantly associated with clinical severity, resulting in a 0.50-point increase in the CGI scores (*p* = 0.0347). These results demonstrate the critical influence of early symptom manifestation on later psychiatric symptom severity, as reflected in both the BPRS and CGI scores, (Figure 1).

### 3.4. Impact of DUP on the BPRS and CGI

Firstly, we assessed the correlation between the DUP and the clinical outcomes of the BPRS and CGI. Th e DUP was highly correlated with both the BPRS (Spearman correlation ρ = 0.97) and CGI (ρ = 0.94). We then investigated the impact of the DUP on clinical severity by categorizing the delay in diagnosis into three groups: no delay (DUP = 0 years), moderate delay (1 < DUP ≤ 5 years), and severe delay (DUP > 5 years). Multivariate regression models were used to evaluate the effects of the DUP on the BPRS and CGI scores, adjusting for potential confounders, as detailed in the statistical methods section.

The multivariable analysis revealed a significant impact of diagnostic delay on both the BPRS and CGI scores, with a more pronounced effect in individuals with a severe DUP. Specifically, patients with no delay had a predicted BPRS mean of 85.53 (95% CI: 76.13 to 94.93). In comparison, patients with a moderate DUP showed a mean increase of 27.16 (95% CI: 19.71 to 34.62), resulting in a BPRS mean of 112.70 (95% CI: 104.92 to 120.47). Patients with a severe DUP had an adjusted BPRS mean of 153.04 (95% CI: 144.42 to 161.67), reflecting a 67.51-point increase (95% CI: 58.54 to 76.48) compared to those with no DUP.

Similar findings were observed for the CGI scores. Patients with no DUP had an adjusted mean of 3.17 (95% CI: 2.73 to 3.61), while patients with a moderate DUP exhibited a 1.11-point increase (95% CI: 0.76 to 1.47), resulting in an adjusted CGI mean of 4.28 (95% CI: 3.92 to 4.64). Patients with a severe DUP showed the largest difference, with a 3.17-point increase (95% CI: 2.75 to 3.59) compared to those with no DUP, leading to an adjusted CGI mean of 4.28 (95% CI: 3.92 to 4.65).

**Figure 2:**
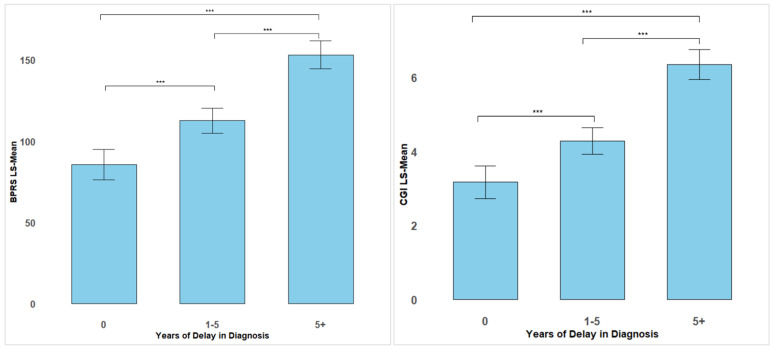
LS means estimates of BPRS and CGI scores stratified by DUP categories. Errors bars represent the 95% CI. ***: *p*-values < 0.001.

However, the interaction between the DUP and the presence of symptoms at 12 years old was not significant, indicating that the DUP impacts the BPRS and CGI scores similarly, regardless of prodromal symptoms at age 12, as shown in Figure 2.

## 4. Discussion

Our study reinforces the evidence that psychotic symptoms before age 12 are strong predictors of greater schizophrenia severity, particularly in terms of negative symptoms and cognitive impairments. Early-onset cases often display profound psychopathology, such as social withdrawal, avolition, and anhedonia, which correlate with worse functional outcomes and illness severity at diagnosis [3,4,26]. These findings align with research by Downs et al. [27], highlighting the prevalence of negative symptoms in early-onset psychosis and their association with treatment resistance. Early prodromal symptoms, such as perceptual disturbances and social withdrawal, also serve as significant predictors of long-term psychopathological outcomes [28].

The onset of psychotic symptoms before age 12 may be influenced by various age- and gender-related factors. For instance, research has shown that early-onset schizophrenia tends to manifest more severely in males, particularly during adolescence, which could impact the nature and progression of prodromal symptoms [29]. Although our study did not specifically explore these differences, the clinical and biological factors contributing to gender differences in psychotic onset could provide valuable insights into the trajectory of prodromal symptoms in children.

Neurodevelopmental disruptions play a critical role in the progression of early-onset psychosis. These factors contribute to heightened cognitive and functional impairments and align with the framework proposed by Owen et al. [30]. Our findings suggest that timely intervention targeting these early symptoms may help prevent long-term decline and alter the trajectory of the disorder.

Furthermore, familial and psychosocial factors may play a critical role in the development of prodromal symptoms. Our study identified a high proportion of patients with a family history of mental illness, which is consistent with the well-established genetic and environmental influences on schizophrenia. Additionally, 71% of participants reported experiencing a stressful event in their history, which may have contributed to the earlier manifestation of psychotic symptoms. This highlights the interaction between genetic predisposition and environmental stressors in shaping the onset of psychosis. Understanding these factors from a developmental perspective is crucial for identifying individuals at risk for schizophrenia at an early stage and may provide further insight into the underlying mechanisms driving the disorder. Future research should aim to investigate these variables more thoroughly to inform preventive strategies and early interventions.

A key finding is the delay between symptom onset and treatment initiation, with a median delay of three years in our sample. A longer DUP strongly correlates with increased symptom severity, as shown by the significant increases in the BPRS and CGI scores. A severe DUP resulted in a 67.51-point increase in the BPRS and in 3.71-point increase in scores compared to cases with no delay. This highlights the critical need to reduce diagnostic delays, as a prolonged DUP exacerbates cognitive deficits and negative symptoms, worsening overall prognosis [31]. The lack of significant interaction between the DUP and early prodromal symptoms suggests that the DUP may be a more consistent predictor of clinical outcomes than the specific timing of symptom onset, reinforcing the importance of early intervention to minimize the DUP across all patients’ profiles and its impact on long-term prognosis.

Our results emphasize the importance of systematic screening for prodromal symptoms, particularly in pediatric populations. Early detection frameworks, such as the PSY-SR, effectively identify key risk factors like interpersonal sensitivity, perceptual distortions, and emerging psychotic symptoms. These tools enable timely intervention, which can delay or prevent psychosis onset in high-risk populations [32]. However, it is important to acknowledge some limitations of the PSY-SR, including its reliance on self-reporting, which may be influenced by factors such as the patient’s insight into their condition and the severity of symptoms. Despite these limitations, the PSY-SR remains a valuable clinical tool for early identification, providing clinicians with structured insight into at-risk individuals. Its use in combination with other clinical assessments could further enhance its accuracy and predictive value in identifying individuals most likely to benefit from early intervention. Interventions such as Cognitive Behavioral Therapy (CBT), family involvement, and integrated treatment models have demonstrated efficacy in reducing the transition to psychosis and improving long-term outcomes [33].

One of the intriguing findings in this study is that adult patients with schizophrenia who exhibited prodromal symptoms before the age of 12 reported using fewer antidepressants compared to those without early prodromal symptoms. Patients with severe schizophrenia in our sample—the ones with prodromal symptoms before 12—often exhibit treatment resistance, and clinicians may focus on optimizing antipsychotic therapy rather than introducing antidepressants, which may not show consistent efficacy in this context [34].

Despite advances in early intervention services, our findings suggest that many individuals remain under-detected during high-risk periods, often due to the subtlety of early symptoms and barriers to accessing care. Enhanced screening protocols for children and adolescents are urgently needed to identify prodromal symptoms of the psychotic spectrum, which frequently precede psychotic episodes.

## 5. Limitations

This study has several limitations that must be considered when interpreting the findings. The retrospective design relies on clinical records and self-reported data, which may introduce recall bias and inconsistencies in symptom timing. The influence of familial factors, such as a mental health history, family dynamics, and psychosocial stressors, could not be assessed in this study, and future research should aim to explore these factors in more depth. Understanding how family systems contribute to the development and severity of schizophrenia could provide valuable insights into early intervention and prevention strategies. Additionally, the sample size limits the generalizability of the findings and the ability to detect subtle subgroup differences, such as the role of specific treatments or nuanced symptom trajectories. Future research should focus on longitudinal studies to examine how early prodromal symptoms evolve and the long-term impact of early interventions. Increasing the sample sizes and integrating real-time clinical data may also enhance the robustness of findings.

## 6. Conclusions

Reducing the duration of untreated psychosis (DUP) is crucial for improving outcomes in psychotic disorders. A longer DUP strongly correlates with greater symptom severity, emphasizing the need for timely detection and intervention. Early diagnosis and minimizing delays are essential, as these modifiable factors significantly impact illness trajectories. From a practical perspective, these findings suggest that mental health professionals, particularly those working in pediatric and adolescent settings, should be equipped with tools such as the PSY-SR to identify early signs of psychosis in at-risk populations. Educators, too, can play an important role by being trained to recognize early behavioral and emotional changes in students, thus facilitating early referrals to mental health services. Furthermore, policymakers should consider developing and implementing public health strategies that promote systematic screening for psychosis in school-aged children. This would ensure that interventions are available to those most in need, particularly in vulnerable groups showing early signs of psychotic spectrum disorders.

Integrating systematic screening into pediatric and adolescent mental health services can accelerate early identification and treatment, reducing the risk of severe symptoms and full-blown psychosis. By targeting DUP reduction and early intervention, clinical settings can improve recovery outcomes and enhance the quality of life for at-risk individuals. Future research should refine detection tools and explore interventions targeting neurodevelopmental pathways to optimize outcomes in psychosis spectrum disorders.

## Figures and Tables

**Table 1:** Socio-demographic and clinical characteristics of the cohort.

	All Cohort (N = 170)	Presence of Prodromal Symptoms Before 12 Years		*p*-Value
		No (*n* = 135)	Yes (*n* = 35)	
Gender (female), *n* (%)	65 (38%)	51 (38%)	14 (40%)	0.8095
Age (years), mean (sd)	50.94 (13.49)	51.32 (14.06)	49.49 (11.05)	0.4753
Marital status, *n* (%)				0.0492
Divorced/widow	13 (8%)	10 (7%)	3 (9%)	
Single	136 (80%)	107 (79%)	29 (83%)	
Married	21 (12%)	18 (13%)	3 (9%)	
Occupational status, *n* (%)				0.0014
Unemployed	64 (38%)	50 (37%)	14 (40%)	
Invalid	26 (15%)	21 (16%)	5 (14%)	
Employed	47 (28%)	39 (29%)	8 (23%)	
Retired	21 (12%)	15 (11%)	6 (17%)	
Student	12 (7%)	10 (7%)	2 (6%)	
Educational level, *n* (%)				0.0101
Elementary school	10 (6%)	2 (6%)	8 (6%)	
Middle school	69 (41%)	3 (9%)	10 (7%)	
High school	78 (46%)	11 (34%)	58 (42%)	
University degree	13 (8%)	16 (50%)	62 (45%)	
Medical History and Drug Utilization				
Experienced stressful event, *n* (%)	120 (71%)	97 (72%)	23 (66%)	0.4776
Familiar history of schizophrenia, *n* (%)	86 (51%)	64 (47%)	22 (63%)	0.1033
Mood Stabilizers, *n* (%)	99 (58%)	78 (58%)	21 (60%)	0.8122
Antidepressant, *n* (%)	22 (13%)	21 (16%)	1 (3%)	0.0494
Antipsychotic, *n* (%)	170 (100%)	135 (100%)	35 (100%)	1.0000
Benzodiazepines, *n* (%)	63 (37%)	54 (40%)	9 (26%)	0.1189

**Table 2:** Clinical outcomes.

	All Cohort (N = 170)	Presence of Prodromal Symptoms at 12 Age		*p*-Value
		No (*n* = 135)	Yes (*n* = 35)	
Age at schizophrenia onset symptoms, median [IQR]	20 [17–26]	20 [17–26]	18 [16–27]	0.2638
Age at baseline schizophrenia evaluation, median [IQR]	25 [20–31]	25 [20–32]	27 [20–31]	0.7765
DUP (years), median [IQR]	3 [1–6]	2 [1–6]	4 [1–10]	0.0726
DUP > 1 year, n (%)	138 (81%)	109 (81%)	29 (83%)	0.7753
CGI, median [IQR]	4 [3–6]	4 [3–6]	5 [3–6]	0.0783
Moderately ill, n (%)	99 (58%)	83 (61%)	16 (46%)	0.0919
Severely ill, n (%)	71 (42%)	52 (39%)	19 (54%)	
BPRS, median [IQR]	113.5 [87–148]	111 [87–145]	131 [89–155]	0.1236
PSY-SR at 12 yo, median [IQR]	33 [13–40]	32 [9–38]	37 [30–44]	0.0002

## Data Availability

Data are available on request. The data are not publicly available due to privacy reasons.
